# Microbiome and its Association with Diseases of the Ear, Nose and Pharynx

**DOI:** 10.2174/0118715303427717251222184941

**Published:** 2026-05-21

**Authors:** Siti Nur Syakinah Md Sabudin, Srijit Das, Norsyamira Aida Mohamad Umbaik, Siti Rosmani Md Zin

**Affiliations:** 1 Medical and Basic Dental Sciences Unit, School of Dental Sciences, Health Campus, Universiti Sains Malaysia, Kubang Kerian 16150, Kota Bharu, Kelantan, Malaysia;; 2 Department of Human and Clinical Anatomy, College of Medicine and Health Sciences, Sultan Qaboos University, Muscat, Sultanate of Oman;; 3 Department of Otorhinolaryngology-Head and Neck Surgery, School of Medical Sciences, Health Campus, Universiti Sains Malaysia, Kubang Kerian 16150, Kota Bharu, Kelantan, Malaysia

**Keywords:** Microbiome, dysbiosis, ENT diseases, drug delivery, pharynx, microorganisms

## Abstract

**Introduction:**

The microbiome refers to the entire community of microorganisms, including bacteria, fungi, viruses, their genes, and the environment that surrounds them. The microbiome is found in the gut, skin, and the oral and nasal cavities, and is an important interface between the human body and the environment outside. Notably, the ear, nose, and throat or pharynx microbiome remains poorly studied. Therefore, the present review discusses the association of the microbiome with various diseases of the ear, nose, and pharynx.

**Methods:**

The literature was obtained by conducting a comprehensive search of the PubMed, Scopus, Web of Science, and Google Scholar databases, followed by screening the abstracts for relevance. The selected articles were then retrieved for deeper exploration.

**Results:**

This review highlights the disturbance of the balance of the microbiome, which is referred to as dysbiosis, that may be the cause of various diseases in the body.

**Discussion:**

We also discuss various microorganisms found in the ear, nose, and pharynx and their link with related diseases and brain health.

**Conclusion:**

The microbiome is important for the better health of the human body as it modulates the immune system. Accordingly, a better understanding of its interaction with the host may lead to advances in drug design.

## 1 INTRODUCTION

Microbiomes are the term used to describe the community of microorganisms, such as bacteria, viruses, and fungi, that exist in a particular environment. In humans, the term specifically refers to the microorganisms that live in certain parts of the body and are essential to human health. In recent decades, researchers’ interest in the significant roles of microbiomes in metabolic, immunological, and neurological processes has increased. Despite numerous studies being conducted, the complexity of microbiomes is still not fully understood [1, 2].

One of the debated aspects in microbiome studies is the definition of a healthy human microbiome. These microbiomes consist of microorganisms that inhabit the skin and mucous membrane of a healthy human being, are present at numerous anatomical sites, and play an important role in preventing the colonisation of pathogens [3]. They are also classified as opportunistic microorganisms that may exhibit their pathogenic potential when there is a weakened immune system or microbiome dysbiosis [4, 5]. Additionally, a single gene transfer can also cause a normally colonised microbiome to become virulent [6, 7].

In recent years, there has been a growing number of microbiome studies that have improved the understanding of disease and health in various human organs. However, the microbiome of the ear, nose, and throat (ENT) or pharynx is scarcely examined. Therefore, this review focuses on the association of microbiomes with diseases of the ENT. To gain a better understanding of the pathologies associated with various ENT diseases, it is essential to enhance our knowledge of the microbiota within the normal ear canal. This insight could lead to the future development of new treatment options, such as modulating the ear microbiome with probiotics or transplanting a healthy microbiome.

Discussion on this topic is crucial as medical problems associated with ENT exert a significant burden on global healthcare, especially hearing impairment [8] and otitis media (OM) [9]. In fact, chronic suppurative otitis media (CSOM), which is also termed active mucosal chronic otitis media, is a leading cause of hearing impairment among children worldwide [10]. This prevalent middle ear infection often involves microbial colonisation [11].

The auditory canal is home to a very complicated ecological network of pathogens and harmless commensal bacteria, fungi, and viruses [12]. The bacteria can be either Gram-positive, Gram-negative, aerobic, or anaerobic. A study conducted by Minami *et al*. (2017) in Japan has analysed the presence of microbiomes in 155 individuals with and without chronic OM recruited from seven medical centres [13]. The most prevalent bacterial phyla on the mucosa of the normal middle ears were Proteobacteria, followed by *Actinobacteria, Firmicutes*, and *Bacteroidetes*. The participants with chronic OM with active inflammation had a lower amount of Proteobacteria and a higher prevalence of *Firmicutes* than the normal middle ears. The study concluded that an alteration of the middle ear microbiome may contribute to the development of chronic OM with active inflammation [13].

An earlier study reported that *Alloiococcus otitis* (*A. otitis)*, *Corynebacterium otitidis*, and *Staphylococcus auricularis* (*S. auricularis)* were the predominant observed microbiomes in the outer ear canal [14]. Moreover, the external ear hosts potential pathogens such as *Streptococcus pneumoniae* (*S. Pneumoniae*) and *Pseudomonas aeruginosa* (*P. aeruginosa*) [15]. Lee *et al*. (2022) have also identified various bacteria in the ear canal of healthy individuals, namely *Staphylococcus* (27.4%), *Cutibacterium* (25.0%), Neisseriaceae (12.6%), and *Corynebacterium* (3.1%). In addition to these bacterial taxa, *S. auricularis*, *Cutibacterium acnes*, *A. otitis*, and *Turicella otitidis* (*T. otitidis*) have been identified in the ear canals of healthy Caucasians [16]. This finding is consistent with a previous study, which also demonstrated the presence of *S. auricularis*, *A. otitis*, and *T. otitidis,* as well as *Propionibacterium acnes* in Caucasian volunteers [17].

The human microbiome is unique to every individual. The discrepancies in findings could be attributed to the differences in host genome, age, race, environmental factors, and the sampled individuals [16]. Different races may have different internal environments in the ear canal. For instance, Africans and Europeans exhibit moist and thick forms of earwax, while Asians have a dry and greyish form of earwax, which contains more protein [18]. Other factors that affect the composition of microbiomes are nutrition and hormones [17].

However, recent investigations highlight that viral members of the ENT microbiome (the virome) can play critical roles in disease pathogenesis. For example, viral upper respiratory tract infections often precede and predispose individuals to bacterial OM by altering the middle-ear environment [19]. Respiratory viruses can induce host epithelial receptors such as ICAM-1 and PAF receptor, facilitating adhesion of bacteria like *S. pneumoniae* to the middle-ear mucosa [19]. Metagenomic analyses have further revealed that diverse viruses (*e.g.*, rhinovirus, coronavirus, bocavirus) are present in most middle ear effusion samples, suggesting that an active virome may contribute to chronic ear inflammation [19]. Virome-focused studies using high-throughput sequencing are now beginning to uncover these viral - bacterial interactions and their mechanisms in ENT diseases, an area previously under-recognised in otolaryngology.

As for fungi at the phylum level, Basidiomycota was the dominant flora inhabiting the normal and infected ear. At the genus level, *Aspergillus* and *Candida* are the candidate pathogens found in healthy sampled individuals [16]. This finding is consistent with a previous study, which reported *Aspergillus niger* and *Candida albicans* as the most common pathogens causing otitis externa (OE) [20]. Studies have demonstrated *Malassezia slooffiae* and *Malassezia restricta* (*M. restricta)* as the most common species, while recent findings showed *Malassezia arunalokei*, *M. restricta*, and *M. globosa* as the predominant species [21-24].

Fungal infections of the ear (otomycosis) are another important dimension of ENT microbiome research. *Aspergillus* species (especially those in the *A. niger* group) and *Candida* yeasts are the predominant otomycosis pathogens, mirroring earlier findings that *A. niger* and *C. albicans* are the most common causes of fungal OE [25]. These fungi can form dense colonisation or biofilms in the external canal, particularly in humid conditions or after antibiotic use, and often require targeted antifungal therapy. Recognising the otic mycobiome’s role is therefore crucial, as antifungal interventions targeting *Aspergillus* and *Candida* are key to managing chronic ear infections where these organisms are implicated.

The microbiotas of the pharynx, including nasopharynx and oropharynx, are also discussed in this review in view of their association with the ear. Disruptions in one site can have cascading effects on the others. For example, dysbiosis in the nasopharyngeal microbiota can lead to the colonisation of the middle ear by pathogens, resulting in OM. Similarly, infections in the oropharynx can spread to the ear *via* the Eustachian tube, highlighting the importance of maintaining a healthy URT microbiota. The immune system plays a central role in mediating these interactions. Commensal bacteria in the URT produce metabolites and stimulate immune responses that help protect against pathogens [26]. Dysbiosis can impair these protective mechanisms, increasing susceptibility to infections [27]. Understanding these complex interactions is critical for developing targeted therapies that restore microbial balance and prevent ear diseases.

## 2 METHODOLOGY

This review employed a narrative literature search to identify studies that have relevant content regarding the microbiome and its association with various ear, nose, and pharynx diseases. The articles obtained were mainly published from 2019 to 2025. Searches were carried out across major scientific databases, including PubMed, Scopus, Web of Science, and Google Scholar. Keywords and their combinations such as “microbiome”, “Ear microbiomes”, “microbial imbalance”, “dysbiosis”, “ear diseases”, “otitis externa”, “otitis media”, “chronic suppurative otitis media”, “pharynx”, “ear, nose and throat”, “brain disease”, “ENT”, “ENT virome”, “microbial biofilm dynamics in otitis media”, “antimicrobial resistance in otitis media”, “fungal infection of ear”, “implications of ear microbiome to brain and mental”, COVID-19 microbiome”, “COVID-19 alteration of microbiome”, “COVID-19 alteration of nasal microbiome”, and “long term nasal dysbiosis due to COVID-19 infection” were applied using appropriate Boolean operators (AND, OR) to optimise the search results. Articles were considered eligible if they investigated the association between microbiome and ear, nose, and pharynx-related diseases, were published in English, and involved human or animal models with accessible abstracts or full texts. Studies were excluded if they were irrelevant to the topic, were non-English, or consisted of non-original works such as editorials, commentaries, or conference abstracts. The titles and abstracts of the articles were first reviewed to determine their relevance. Articles that appeared suitable were then selected for full-text reading. These selected articles were examined in more detail to extract important information related to the study. This manuscript was written entirely by the authors.

## 3 EAR DISEASES AND THE MICROBIOTA ASSOCIATED WITH EACH DISEASE

Microbial community composition of the ear changes as it transforms from a healthy to diseased state [28, 29]. Earlier studies showed the presence of otopathogens in the middle ear fluid of patients with acute OM (AOM), namely *S. pneumoniae*, *Haemophilus influenzae (H. influenzae)*, and *Moraxella catarrhalis* (*M. catarrhalis)* [30]. (Fig. **1**) More recent studies have demonstrated that *P. aeruginosa* is the dominant pathobiont responsible for the development of CSOM due to its advanced survival strategies [31-33]. In CSOM, both *P. aeruginosa* and *Staphylococcus aureus* (*S. aureus*) commonly persist by forming robust biofilms on the middle-ear mucosa. These biofilms shield bacteria from antibiotics and host immunity, leading to recalcitrant infection and ongoing inflammation [34]. A middle-ear biofilm is now recognised as a key driver of CSOM pathology, often containing multiple organisms encased in an extracellular matrix, which complicates eradication. *P. aeruginosa* does not always cause CSOM in certain individuals, which could be due to the person’s ability to withstand or adapt to the middle ear microbial community changes [34]. This innate capacity for survival prevents it from falling into an imbalanced or diseased state [35]. However, variations in individual variability to microbial immune evasion might also be influenced by the varieties of bacterial strains and virulence in the population [34].

The internal and middle ear are usually sterile [36, 37]. However, dysbiosis can result in negative health effects, including inflammation, infections, or disruptions in normal bodily processes [28, 38-41]. This is consistent with a previous study, which has demonstrated significant up-regulation of inflammatory cytokines in the CSOM group [42].

Since dysbiosis of the middle ear both results from and contributes to inflammation, modifying immune responses could help restore microbial balance and reduce tissue damage [34]. This was demonstrated by a previous study, in which the knockdown of Nuclear erythroid 2-related factor (Nrf2) reversed the chronic inflammation and attenuated CSOM by upregulating Toll-like receptor 4 (TLR4) [42]. This study found that the levels of Nrf2 and TLR4 showed negative correlations in CSOM clinical tissues. This finding suggests that TLR4 could be targeted by Nrf2 during the progression of CSOM [42]. TLR4 is a crucial activator of innate immunity that helps fight against pathogens and thus prevents infection [43]. Interleukin (IL)-1 receptor antagonists and NLRP3 inhibitors have also shown the ability to reduce inflammation and slow the progression of inflammatory disease [44, 45].

Another microbiome-associated ear disease is the acute OE (AOE), a condition characterised by cellulitis of the ear canal skin and subdermal tissue [46]. The External Auditory Canals (EAC) are normally protected against environmental pathogens by the overlying skin, which is firmly attached to the bone and cartilage, and is connected to the eardrum medially. The homeostasis of the skin epithelium is also maintained by the commensal organisms that colonise the skin surface [47]. *Corynebacterium* and *Staphylococcus* species have been reported to contribute to the prevention of infections by competing with pathogens and maintaining an acidic environment [17]. However, the EAC skin can be exposed to infection when the equilibrium of pathogenic and non-pathogenic strains is disrupted. The disruption or shift in microbial composition may provide an opportunity for the growth of harmful species, which can cause skin inflammation [48, 49].

A study in North America reported that most OE cases are caused by bacterial infection [50]. Previous studies have reported *P. aeruginosa* and *Staphylococcus* species as the frequently isolated pathogens in acute external otitis [46, 51, 52]. These organisms thrive in moist environments and can form biofilms, making infections difficult to treat [53]. These commensals may affect the epithelial barrier and innate immunity in the environments of the EAC, where the microbiome composition is disrupted.

Some of the factors that may affect microbiome composition include host genome, family history, gender, age, nutrition, and hormones. Wet or dry cerumen type can also influence the microbiome composition. Dry-type cerumen is prevalent among East Asian populations, whereas Europeans and Africans commonly have wet-type cerumen [18].

Endogenous infections can also develop in some people in the presence of predisposing factors such as blockage of the EAC, exostoses, or excess hair on the EAC. Other additional factors include itchy skin, wearing hearing aids or earplugs, using cotton buds, foreign bodies, swimming, and stress [54-56].

Findings from a previous study showed the significance of the microbiome in the development of acute OE, which warrants further studies on interactions between humans and the microbiome. Recent studies have highlighted the potential of microbiome-based therapies, such as topical probiotics, to restore the EAC microbiota and prevent recurrent infections [57]. Further understanding of this matter may help develop novel drug delivery systems that selectively target immune cells [34]. In fact, a previous study suggested the consumption of oral probiotics to suppress the ongoing inflammation and restore a balanced microbiome composition [58]. More information from future studies may also help improve diagnostic tools that facilitate early detection and contribute to prevention strategies [34].

Another critical concern in chronic OM (especially CSOM) is antimicrobial resistance due to long-term or repeated antibiotic therapy. Chronic ear infections often necessitate multiple courses of antibiotics, which can select for resistant strains. For instance, recent microbiological studies of CSOM cases have shown high rates of resistance in the predominant pathogens: over 90% of *P. aeruginosa* isolates were resistant to common β-lactams (*e.g.*, cefuroxime or amoxicillin-clavulanate), and *S. aureus* isolates frequently exhibited resistance to penicillins and even macrolides, with one report noting >90% of *S. aureus* in CSOM resistant to erythromycin [59]. In some settings, a substantial subset of CSOM staphylococcal isolates is MRSA, further complicating treatment. Such patterns of multi-drug resistance in chronic ear infections pose a therapeutic challenge, as standard empirical medications may be ineffective. These findings underscore the need for culture-directed therapy in CSOM and judicious use of antibiotics. They also motivate exploration of alternative treatments, for example, topical antiseptics, antibiotic-impregnated gels, or adjunctive anti-biofilm agents to manage chronic OM infections when conventional antibiotics fail. Monitoring local resistance trends and implementing antibiotic stewardship in chronic ENT infections are therefore crucial steps to prevent a “pre-antibiotic era” relapse in OM management.

## 4 THROAT (PHARYNX) MICROBIOTA ASSOCIATED WITH THE EAR

The pharynx (throat) consists of three parts: the nasopharynx, oropharynx, and laryngopharynx. The nasopharynx, located behind the nose and above the soft palate, connects the nasal cavity to the upper pharynx and contains the Eustachian tube opening, linking the ear and nose. The oropharynx, situated behind the mouth, extends from the soft palate to the epiglottis and includes the tonsils and back of the tongue. The laryngopharynx, found behind the larynx, leads to the oesophagus and trachea [60]. We focus on the microbiome of the nasopharynx and oropharynx and their association with ear disease. Nasopharynx and oropharynx are also parts of the human Upper Respiratory Tract (URT) [61], which harbours a diverse and dynamic microbial community [62, 63]. This microbiota plays a crucial role in maintaining homeostasis, protecting against pathogens, and modulating immune responses. Recent advances in sequencing technologies have enabled researchers to explore the complex interactions between the URT microbiota and ear health, particularly in the context of diseases such as OM and OE.

### 4.1 Nasopharyngeal Microbiome

The nasopharyngeal microbiome is the community of microorganisms residing in the upper part of the pharynx behind the nose and plays a pivotal role in both the development and prevention of ear diseases, particularly OM. The Pathogen Reservoir Hypothesis (PRH) posits that the nasopharynx, including the adenoid pad, serves as a primary reservoir for bacterial pathogens that can ascend to the middle ear *via* the Eustachian tube, leading to OM [64].

Otitis media, a common middle ear infection, particularly in children, has been strongly linked to disruptions in the nasopharyngeal microbiota. Research consistently shows that the nasopharyngeal microbiota of children with AOM differs significantly from that of healthy children [65, 66]. During an AOM episode, the overall bacterial density in the nasopharynx tends to be lower, but otopathogens often become dominant over commensal bacterial families [64]. An unstable nasopharyngeal microbiota during an upper respiratory tract infection (URTI), characterised by a predominance of otopathogens, is associated with a higher risk of the URTI progressing to AOM [64]. Interestingly, the alpha and beta diversity of the nasopharyngeal bacterial microbiota in children with AOM show a clear age dependency, with older children generally having higher richness and more personalised bacterial profiles [66].

OM-prone individuals often exhibit dysbiosis (Fig. **2**), characterised by reduced microbial diversity and the absence of commensal bacteria, such as *Dolosigranulum* and *Corynebacterium*, both of which are associated with an increased risk of OM [67, 68]. These commensals are nonpathogenic taxa of the nasopharynx [30, 69] and are thought to play a protective role by competing with pathogens and modulating immune responses. Previous studies have shown that the nasopharyngeal microbiota of children with recurrent OM is often dominated by pathogens such as *S. pneumoniae*, *H. influenzae*, and *M. catarrhalis* [62, 70]. These pathogens can ascend the Eustachian tube and colonise the middle ear, leading to infection and inflammation. The use of antibiotics, while effective in treating AOM, can further disrupt the nasopharyngeal microbiota, potentially predisposing individuals to recurrent infections [71].

The nasopharyngeal microbiota undergoes a maturation process with age, with density and diversity stabilising in early adulthood [72]. Age is a significant risk factor for OM, with infants and young children being more susceptible. Environmental factors such as daycare attendance can influence the nasopharyngeal microbiota and potentially increase the risk of OM [73]. Antibiotic exposure, particularly in early childhood, can significantly alter the nasopharyngeal microbiota, reduce beneficial commensals, and potentially increase the risk of recurrent infections [74].

Interestingly, certain commensal bacteria exert protective effects by inhibiting pathogen growth *via* secretion of antimicrobial enzymes, metabolic byproducts, or competition for attachment sites. Therefore, modulating the nasopharyngeal microbiota through probiotics is being explored as a potential strategy for preventing and treating ear infections [68, 75]. Some studies suggest that certain *Lactobacillus* and *Streptococcus* strains may reduce the incidence of AOM [76]. For instance, *Streptococcus salivarius* (*S. salivarius*) has shown promise in reducing recurrent AOM episodes and clearing middle ear effusion (Jörissen *et al*., 2021) [77]. Besides, *Staphylococcus epidermidis* secretes a serine protease, Esp, which degrades *S. aureus* biofilms and hinders *S. aureus* nasal colonisation *in vivo* [78]. In the nasopharynx, *Dolosigranulum pigrum* and *Corynebacterium pseudodiphtheriticum* have been shown to synergistically reduce *S. pneumoniae* growth when co-cultured, suggesting a protective antagonistic effect [79]. In addition, a higher abundance of *Corynebacterium* species in the upper airway is inversely correlated with *S. pneumoniae* colonisation, and some *Corynebacterium* strains directly inhibit *S. pneumoniae* growth *in vitro* [80]. Such interbacterial competition and inhibition likely contribute to microbial homeostasis and help prevent the overgrowth of pathogens in the ENT region.

### 4.2 Oropharyngeal Microbiome

The adenoid (lying on the roof and posterior wall of the nasopharynx) and the palatine tonsils (located in the tonsillar fossa of the oropharynx) are physiologically complex mucosa-associated lymphoid tissues that contribute to the formation of Waldeyer’s ring [81]. Adenoid or pharyngeal tonsil has been demonstrated by Fagö-Olsen *et al*. (2019) to contain the microbiomes, namely *S. pneumoniae*, *H. influenzae*, and *M. catarrhalis,* which indicate that adenoids and not the palatine tonsils are the main reservoir for potential pathogens for AOM and secretory OM (SOM) in children [82].

In addition, emerging evidence indicates that the oropharyngeal microbiota can influence the effectiveness of treatments for ear infections. Several mechanisms have been proposed to explain how the oropharyngeal microbiota can influence the development and progression of ear diseases. One of the primary routes is the ascension of pathogenic bacteria from the oropharynx and nasopharynx, through the Eustachian tube, into the middle ear [83]. The continuous flux of saliva to the oropharynx facilitates aerosol formation and the transfer of oral bacteria to the middle ear during the intermittent Eustachian tube opening [84].

Streptococcal species are among the prominent members of oral microbiomes that disperse in the aerodigestive tract and enter the middle ear during intermittent openings of the Eustachian tube [85]. The otic streptococci are commonly associated with *S. salivarius* and *Streptococcus parasanguinis,* which are the early commensals of the human oral cavity after birth [86, 87]. In addition to these bacteria, the oropharyngeal commensals that transiently spread in the aerodigestive tract and the middle ear *via* saliva aerosols include *Neisseria perflava, Neisseria subflava,* and *Neisseria flavescens* [84, 88].

Probiotic interventions aimed at restoring a healthy oropharyngeal microbiota are being explored to prevent recurrent infections and improve treatment outcomes. A previous study reported that probiotics may reduce the incidence of AOM in children not prone to such infections [89]. In line with this, a study by Cárdenas *et al*. (2019) demonstrated that administering *Lactobacillus salivarius* PS7 significantly decreased episodes of recurrent AOM in children [90]. These findings suggest that targeting the oropharyngeal microbiota through probiotics could be a promising strategy for managing ear infections.

## 5 COVID-19/SARS-CoV-2 AND ALTERATION OF THE UPPER AERODIGESTIVE TRACT MICROBIOME

During the era of the coronavirus disease 2019 (COVID-19) pandemic, the striking features that were observed were that the infection was mild in some individuals and severe in others, which ultimately led to death. The reason why this occurred was unknown, but it was found to be partly related to the composition of nasal and upper airway microbiota. Nasal microbiota dysbiosis was found to play a role and has a strong correlation in determining the severity of COVID-19 infection in an individual [91]. The COVID-19 infection also altered the composition of normal human respiratory tract microbiota by decreasing the numbers and evenness of distribution of the microbiota. It was observed that the COVID-19 infection led to increased abundance of common nasal opportunistic species, including *Citrobacter koseri, Dolosigranulum pigrum, H. influenzae, Klebsiella pneumoniae,* and *M. catarrhalis* [92]. A meta-analysis also reported an increased number of *Streptococcus* and *Neisseria* sp in COVID-19 patients’ oral microbiota, leading to co-infection and thus more severe symptoms [93]. Alterations in the respiratory microbiome, as identified through metagenomic profiling, demonstrate an increase in opportunistic pathogens, including *S. pneumoniae, S. aureus,* and *H. influenzae,* and a concurrent decrease in commensal bacteria such as *Prevotella, Veillonella,* and *Bacteroides* [94]. Furthermore, the dysbiosis of upper respiratory tract microbiota has significantly correlated with the concentration of proinflammatory cytokines and mortality [95]. This microbiota dysbiosis in COVID-19 infection not only occurs in the upper respiratory tract but is also evident in gut and skin microbiota, leading to systemic activation of inflammatory pathways and immune dysregulation [96]. Notably, upper respiratory tract dysbiosis often persists even after the COVID-19 infection and its clinical manifestations have subsided, which may explain the pathogenesis of long COVID and chronic fatigue syndrome [97, 98].

## 6 MICROBIOTA-EAR-BRAIN INTERACTION AND ITS IMPLICATIONS

Recent studies suggest that, beyond local ENT diseases, the ear microbiome (Table **1**) may influence brain function and mental health through inflammatory and neural pathways. Mounting evidence indicates that dysbiosis of the EAC and middle-ear microbiota can activate neuroimmune signalling and sensory circuits connecting the ear and brain [99-101].

In a case–control study, Feng *et al*. (2023) [99] reported that participants with Generalised Anxiety Disorder (GAD) exhibited greater microbial diversity in the EAC, characterised by a significant increase in *Proteobacteria* and a decrease in *Firmicutes*, compared with age- and sex-matched controls. The relative abundance of *P. aeruginosa* was markedly elevated in GAD patients and correlated positively with Dizziness Handicap Index (DHI) scores, implying that otic dysbiosis may induce vestibular dysfunction contributing to anxiety.

The biological plausibility of this “microbiota–ear–brain axis” is reinforced by mechanistic data showing that chronic ear inflammation elevates IL-1β, IL-6, and TNF-α, cytokines capable of crossing the blood–brain barrier or signalling *via* afferent cranial nerves to the central nervous system (CNS) [102]. These cytokines trigger microglial activation, alter neurotransmitter metabolism, and are implicated in anxiety, depression, and cognitive change. In COM, persistent infection and biofilm activity from *P. aeruginosa* and *S. aureus* sustain inflammation and cytokine release. Consistently, patients with COM experience higher depression, anxiety, and stress scores, proportional to hearing loss and disease severity [103].

The vestibular–limbic pathway further links ear pathology to emotional regulation. The vestibular nuclei connect directly with the amygdala, hippocampus, and insular cortex regions, modulating arousal and mood. Meta-analytic evidence demonstrates that adults with vestibular disorders, often sequelae of chronic ear disease, have a significantly increased risk of anxiety and depressive disorders [104]. Thus, inflammatory or dysbiotic insults within the middle or inner ear may perturb vestibular signalling to limbic centres, amplifying psychological symptoms.

In addition to psychiatric associations, hearing loss secondary to recurrent OM has been linked to cognitive decline and dementia. A longitudinal cohort study found that even mild hearing loss accelerated cognitive deterioration and increased dementia risk [105]. Although these studies did not directly assess microbial composition, persistent ear dysbiosis may promote both inflammatory and sensory-deprivation mechanisms underlying neurocognitive decline.

Another neurodevelopmental link involves Autism Spectrum Disorder (ASD), a condition marked by social and communication deficits and repetitive behaviours. Children with ASD show higher rates of both serous and suppurative OM [106]. Early-life ear infections, possibly accompanied by microbiome disruption, are thought to influence language acquisition and social development [107]. Immune activation during critical periods of neurodevelopment may alter microglial maturation, thereby shaping neural circuit formation.

Collectively, these findings indicate that the ear microbiota–brain connection operates through multiple interacting pathways:

1. Otic dysbiosis and infection leading to local cytokine release,
2. Neuroimmune communication *via* circulating cytokines and neural afferents,
3. Vestibular and auditory disturbance affecting limbic and cortical circuits, and
4. Neuroinflammation and sensory deprivation contribute to anxiety, depression, and cognitive decline.

Despite growing evidence, current literature on the microbiota-ear-brain axis remains limited. Future longitudinal and mechanistic studies integrating metagenomics, cytokine profiling, and neuroimaging are needed to establish causality and identify microbial or immune biomarkers predictive of neurological outcomes [108].

## 7 HUMAN MICROBIOME PROJECT

Large-scale initiatives such as the National Institutes of Health (NIH) Human Microbiome Project (HMP) and the Integrative Human Microbiome Project (iHMP) have profoundly expanded our understanding of microbial diversity and function across major human body sites, including the oral cavity, nasal passages, gastrointestinal tract, skin, and vagina [111, 112]. These landmark efforts have established comprehensive microbial reference profiles and elucidated host–microbiome interactions in health and disease such as prediabetes, inflammatory bowel disease, and pregnancy. However, despite this broad scope, the ear has not been directly included as a distinct microbial habitat in either the HMP or iHMP frameworks, although nearby skin regions behind the ear were sampled [113]. This exclusion underscores a significant gap in our current microbiome reference datasets. Recognising and addressing this omission is essential for completing the picture of the human microbiome within the ENT axis and for deepening our understanding of microbial contributions to ENT-related diseases.

## CONCLUSION

There is a paucity of studies on the ENT microbiome. The microbiome plays an important role in human health, as dysbiosis has been associated with various diseases in the human body. It is important to recognise the various organisms associated with ENT diseases, as they can define the progression of the disease and its treatment outcomes. According to the microbes associated with each ENT disease, targeted therapy may be beneficial. The use of probiotics could help in the restoration of any microbial imbalance. Utilising antibiotics against ENT pathogens can also disrupt microbiomes. Considering the adverse effects of many synthetic drugs, newer natural product-based drugs could also be designed. There is a need to develop the latest next-generation DNA sequencing techniques and place more emphasis on metagenomic studies. A holistic approach could benefit diagnostic sensitivity and help in targeted treatment and better drug design.

## Figures and Tables

**Fig. (1):**
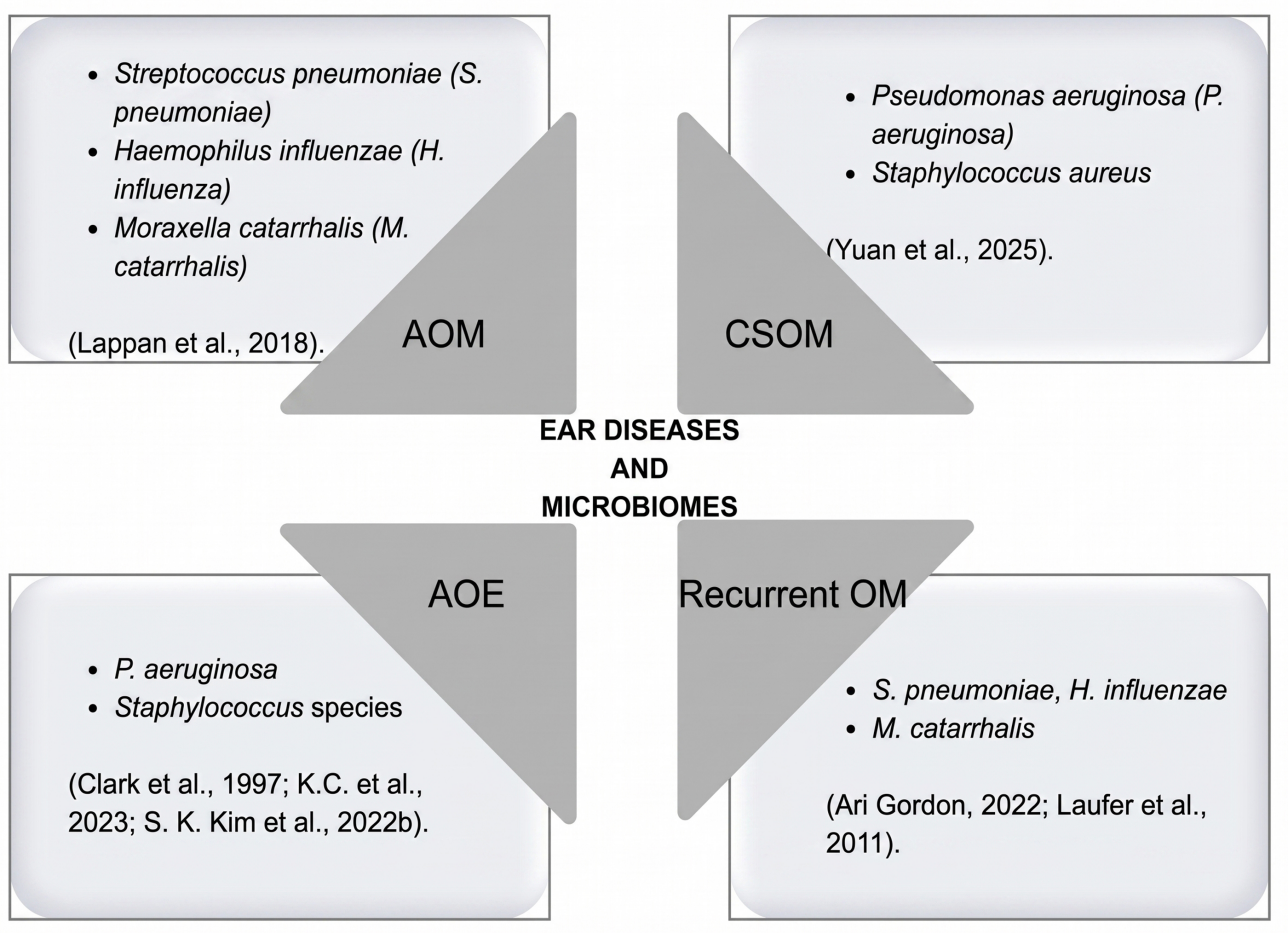
Ear diseases (AOM: Acute otitis media; CSOM: Chronic suppurative otitis media; AOE: Acute otitis externa) and their associated microbiomes.

**Fig. (2):**
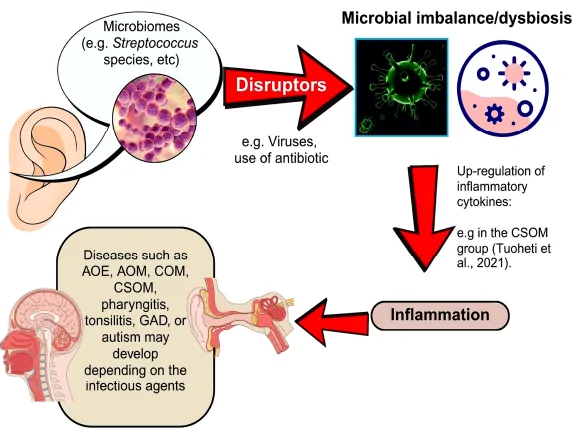
Microbiomes and ear-related diseases (AOE: Acute otitis externa; AOM: Acute otitis media; COM: Chronic otitis media; CSOM: Chronic suppurative otitis media; GAD: Generalised anxiety disorder)-Drawn with Canva software.

**Table 1:** Microbiomes associated with ENT.

**Region**	**Microbiome**	**References**
External ear	*Staphylococcus auricularis*, *Cutibacterium acnes*, *Alloiococcus otitis*, *Turicella otitidis*, *Propionibacterium acnes*, *Corynebacterium otitidis, Streptococcus pneumoniae* (*S. Pneumoniae*) or *Pseudomonas aeruginosa*, *Aspergillus niger, A. fumigatus, Candida albicans, Candida tropicalis, Malassezia slooffiae, Malassezia restricta, Malassezia arunalokei*, *Malassezia globosa*	[14-17, 20-24, 52, 109]
Middle ear	*S. pneumoniae*, *H. influenzae*, *Moraxella catarrhalis (M. catarrhalis)*, *P. aeruginosa, Staphylococcus aureus, Stephanoascus ciferrii* (also called *Candida ciferrii* or *Trichomonascus ciferrii*)	[30-32, 62, 110]
Nasopharynx	*S. pneumoniae, Haemophilus influenzae (H. influenza),* and *M. catarrhalis*.	[62, 70]
Adenoid/ Pharyngeal Tonsil	*S. pneumoniae, H. influenzae,* *M. catarrhalis*	[82]
Oral cavity	*Streptococcus salivarius, Streptococcus parasanguinis*	[86, 87]
Oropharynx	*Neisseria perflava, Neisseria subflava, Neisseria flavescens*	[84, 88]

## STUDY LIMITATIONS

The limitation of this review is the scarcity of recent studies published from 2020 onwards that discuss the ear microbial community, including the virome, and its associations with otorhinolaryngology- and brain-related diseases.

## LIST OF ABBREVIATIONS

AOE: Acute Otitis Externa
AOM: Acute Otitis Media
COVID-19: Coronavirus Disease 2019
DNA: Deoxyribonucleic Acid
ENT: Ear, Nose, and Throat
CSOM: Chronic Suppurative Otitis Media
HMP: Human Microbiome Project
IHMP: Integrative Human Microbiome Project
OM: Otitis Media
OE: Otitis Externa
NRF2: Nuclear Erythroid 2–Related Factor
TLR4: Toll-Like Receptor 4
IL-1: Interleukin-1
NIH: National Institutes Of Health
NLRP3: NOD-Like Receptor Family, Pyrin Domain Containing 3
EAC: External Auditory Canal
GAD: Generalised Anxiety Disorder
DHI: Dizziness Handicap Index
URT: Upper Respiratory Tract
PRH: Pathogen Reservoir Hypothesis
URTI: Upper Respiratory Tract Infection
